# Incidence trends, histological subtypes, and topographical distribution of bladder cancer in Iran: a study based on the Iranian National Cancer Registry during 2006-2015

**DOI:** 10.3389/fonc.2024.1423968

**Published:** 2024-10-08

**Authors:** Alvand Naserghandi, Mehdi Azizmohammad Looha, Melika Jameie, Zeynab Moradian Haft Cheshmeh, Kosar Namakin, Najmeh Golmakani, Aydin Feyzi, Hadis Shabanipour, Mohammad Amin Tofighi Zavareh, Farzad Allameh, Mohammad Esmaeil Akbari

**Affiliations:** ^1^ Student Research Committee, Shahid Beheshti University of Medical Sciences, Tehran, Iran; ^2^ Basic and Molecular Epidemiology of Gastrointestinal Disorders Research Center, Research Institute for Gastroenterology and Liver Diseases, Shahid Beheshti University of Medical Sciences, Tehran, Iran; ^3^ Neuroscience Research Center, Iran University of Medical Sciences, Tehran, Iran; ^4^ Iranian Center of Neurological Research, Neuroscience Institute, Tehran University of Medical Sciences, Tehran, Iran; ^5^ Department of Epidemiology, School of Public Health, Iran University of Medical Sciences, Tehran, Iran; ^6^ Student Research Committee, Mashhad University of Medical Sciences, Mashhad, Iran; ^7^ Student Research Committee, School of Nursing and Midwifery, Shahid Beheshti University of Medical Sciences, Tehran, Iran; ^8^ Men’s Health & Reproductive Health Research Center, Shahid Beheshti University of Medical Sciences, Tehran, Iran; ^9^ Cancer Research Center, Shahid Beheshti University of Medical Sciences, Tehran, Iran

**Keywords:** bladder cancer, incidence, histology, topography, registry, Iran, urinary bladder neoplasms

## Abstract

**Background:**

Bladder cancer (BCa) is a significant public health concern. This study aimed to analyze the incidence trends, histological subtypes, and topographical distribution of BCa in Iran over a decade.

**Methods:**

This retrospective registry-based study analyzed data on BCa patients diagnosed between March 20, 2006, and March 20, 2015. Following data quality control, age-standardized incidence rates (ASIRs) were calculated for BCa overall, by sex and histological subtype using the new World Health Organization (WHO) standard population.

**Results:**

We identified 51,379 BCa cases (81.97% male) with a mean age of 65.10 ± 14.89 years. The overall ASIR was 8.92 per 100,000 (95% CI: 8.84-9.00). While a modest increase in ASIR was observed overall (8.77 in 2006 to 9.64 in 2015) and among males (14.13 in 2006 to 15.95 in 2015) during the study period, males consistently had a significantly higher ASIR compared to females (approximately 4.5:1 ratio). BCa incidence showed a progressive increase across older age groups, particularly those aged 40-44 to 80-84 years. Urothelial cell carcinoma (UCC) was the most prevalent histological type (ASIR: 8.19 to 7.93), followed by adenocarcinoma (AC; ASIR: 0.13 to 0.11). Squamous cell carcinoma (SCC) displayed a decreasing trend (ASIR: 0.11 to 0.06). Both UCC and AC were more frequent in males (approximately 5 and 3 times higher than females, respectively). Malignant neoplasm of the bladder, unspecified, constituted over 95% of BCa topography classifications.

**Conclusion:**

This study identified a modest rise in BCa incidence, with male predominance and a higher burden in older adults. Further investigation into potential risk factors contributing to this increase is warranted.

## 1 Introduction

Bladder cancer (BCa) is the ninth most prevalent malignancy on a global scale, causing 614,000 new incidences in 2022. Among these, 472,000 diagnoses occur in males, while 142,000 occur in females. Incidence rates are notably elevated among males, registering at 9.3 cases per 100,000 individuals, while in females, the rate is considerably lower at 2.4 per 100,000 persons. This renders BCa the sixth most prevalent cancer in males and the eighteenth most common in females (1). In Iran, the incidence rate of BCa was 11.50 in 2020, with males exhibiting a rate of 20.30 and females 3.44 per 100,000 persons, respectively (2).

Globally, the trend in BCa incidence rates remained relatively constant from 1990 to 2019, according to the Global Burden of Disease (GBD) study 2019. However, an upward trend was observed in Southeast Asia, East Asia, Oceania, Central Europe, Eastern Europe, Central Asia, North Africa, and the Middle East (3). Moreover, predictions indicate that by 2040, the annual number of new BCa cases will increase to 991,000, representing a 72.8% increase from 2020. Notably, recent studies in Iran have shown an increasing trend in BCa incidence rates during 2014-2017 (4) and 2005-2020 (2). This rise has been attributed to various factors, including opium use, smoking, being male sex (5), co-exposure to arsenic-contaminated drinking water and tobacco smoking (6), occupations involving metal processing and exposure to aromatic amines (7, 8), waterpipe smoking (9), as well as dietary habits characterized by high consumption of trans-fatty acids, polyunsaturated fatty acids, total fat, saturated fatty acids, sodium, and cholesterol (10).

Beyond incidence rates, the heterogeneous pathology of BCa plays a critical role in guiding treatment decisions and influencing patient outcomes. Different BCa histomorphologies (microscopic appearances) exhibit distinct clinical behaviors, impacting diagnosis, treatment response, and prognosis (11). According to a database called the “Surveillance, Epidemiology, and End Results” (SEER) program for 1990-2007, the most common types of BCa were urothelial cell carcinoma (UCC), squamous cell carcinoma (SCC), adenocarcinoma (AC), sarcoma, small cell carcinoma, signet ring carcinoma, and spindle cell carcinoma, respectively (12). In Iran, UCC was the predominant histological type of BCa, with a decreasing trend in incidence rates from 2003 to 2008 (13).

Despite existing research on BCa incidence and histology in Iran, a comprehensive investigation encompassing both aspects over an extended period is lacking. This study aims to address this gap by analyzing BCa incidence trends in Iran from 2006 to 2015, stratified by sex, age, histological subtypes, and topography providing valuable insights into the epidemiology and pathology of BCa in the region.

## 2 Methods

### 2.1 Study design

The current investigation comprises a registry-based retrospective cohort study aimed to evaluate BCa incidence trends in Iran, stratified by sex, age, histological subtypes, and topography.

### 2.2 Setting

Data for this study was obtained from the Iran National Cancer Registry (INCR), a nationwide registry maintained by the Ministry of Health and Medical Education (MoHME) in Iran. The INCR collects comprehensive clinical information on individuals diagnosed with cancer between March 20, 2006, and March 20, 2015. This dataset includes detailed records of clinical procedures, pathological diagnoses, and death certificate (DCO) information. Data collection is overseen by all 60 medical universities affiliated with the MoHME, ensuring nationwide coverage across all Iranian provinces. The INCR integrates information from pathology reports and clinical/paraclinical data obtained from hospitals. Due to the inherent time lag between cancer diagnosis and data entry, this study employed the most recent complete dataset available at the time of analysis, acknowledging the absence of real-time data updates.

### 2.3 Participants

Participants in this study were patients with BCa registered in the INCR. Only patients with confirmed malignant bladder tumors, classified under malignant behavior codes (/3) according to the International Classification of Diseases for Oncology (ICD-O3) (14), were included. Patients with benign or tumors of uncertain behavior were excluded. For patients with multiple primary tumors, only distinct primary tumors, confirmed based on pathology and clinical judgment, were included, while recurrences and metastases were excluded. Additionally, patients with incomplete or inaccurate data (7%) were excluded from the analysis following data quality control procedures.

### 2.4 Data quality control

To ensure data quality, a multi-step process was implemented. The INCR’s existing data quality measures were employed to address potential inconsistencies in topography and morphology information. Accordingly, discrepancies were identified and subsequently re-evaluated, with inaccurate entries excluded. Additionally, tumor types were scrutinized to verify the use of appropriate BCa diagnostic procedures. Patient demographics (age and date of birth) were also verified, with corrections or exclusions made for errors. Duplicate entries were identified through a comprehensive examination of demographics, including first and last names, sex, and father’s name. Exact matches were flagged and removed from the dataset. To further validate diagnoses, a random sample (20%) of patients underwent telephone interviews to confirm BCa type and gather additional information.

### 2.5 Variables

The primary outcome of interest was a confirmed diagnosis of BCa based on pathology (or cytology), clinical outcomes, and DCO records.

Patient information related to the topography and histology of BCa was categorized according to the third edition (first revision) of the ICD-O3. This included BCa of all types (topography codes C67.0-C67.9), exhibiting malignant behavior (/3 behavior code). BCa histology was further classified into common cell types: urothelial carcinoma (UCC; codes 8120, 8130), squamous cell carcinoma (SCC; codes 8070, 8076), adenocarcinoma (AC; codes 8140, 8144, 8255, 8260, 8263, 8310, 8323, 8480, 8481, 8570, 8574, 8575), and others (sarcoma, small cell carcinoma, signet ring carcinoma, spindle cell carcinoma) (14).

Patient demographics including sex, age, date of birth, and city of residence were collected. National census data from the “Statistical Center of Iran” provided population estimates for calculating BCa incidence rates across different age groups for the years 2006, 2011, and 2016 (15). Since population data for other years was unavailable, these were estimated using the growth rate between consecutive censuses (16).

### 2.6 Statistical analysis

Crude incidence rates, expressed per 100,000 person-years, and their corresponding 95% confidence intervals (CIs) were computed for each age group as follows:


(1) $${\text{a}}_{i}=\text{Crude Incidence rate}=\frac{\text{Number of new cases of disease}}{\text{Population at risk}}\text{in a period of time}$$

(2) $$\text{s}.\text{e}.({\text{a}}_{i})=\begin{array}{c} \sqrt{100000^{2}\times \frac{1}{\text{n}}\times \frac{\text{r}}{n}\times \left(1-\frac{\text{r}}{n}\right)}\\ {\text{a}}_{i}\pm {\text{Z}}_{\alpha /2}\times \text{s}.\text{e}.({\text{a}}_{i}) \end{array}$$

The calculation process involved defining “r” as the number of cases occurring in the i^th^ age class, and “n” as the person-years of observation in the age class during the same period when cases were counted. Age-standardized incidence rates (ASIRs) per 100,000 and their associated 95% CI were determined by employing the new World Health Organization (WHO) standard population (2000-2025), treating these estimations as weights in the standardization method. The formulas for these calculations are represented as (17, 18):


$$\text{ASIR}=\frac{\sum_{\text{i}=1}^{\text{A}}{\text{a}}_{\text{i}}{\text{w}}_{\text{i}}}{\sum_{\text{i}=1}^{\text{A}}{\text{w}}_{\text{i}}}$$

(3) $$\text{Var}(\text{ASIR})=\frac{\sum_{\text{i}=1}^{\text{A}}\left[{\text{a}}_{i}{\text{w}}_{i}^{2}(100000-{\text{a}}_{i})/{\text{n}}_{i}\right]}{\left(\sum_{\text{I}=1}^{\text{A}}{\text{w}}_{i}\right)}$$

$$\text{s}.\text{e}.(\text{ASIR})=\sqrt{\text{Var}(\text{ASIR})}$$

$$\text{ASIR}\pm {\text{Z}}_{\alpha /2}\times (\text{s}.\text{e}.(\text{ASIR}))$$

Here ${\text{Z}}_{\alpha /2}$ denotes a standardized normal deviation, a_i_ represents the crude incidence rate in each age category, and w_i_ is the new WHO standard population. By applying a direct method, the standardized rate ratio (SRR) and 95% CI were obtained (19) as follows:


$${\left(\text{ASI}{\text{R}}_{1}/\text{ASI}{\text{R}}_{2}\right)}^{1\pm ({\text{Z}}_{\alpha /2}/\text{x})}$$

(4) $$\text{X}=\frac{\text{ASI}{\text{R}}_{1}-\text{AS}{\text{R}}_{2}}{\sqrt{\text{s}.\text{e}.{\left(\text{ASI}{\text{R}}_{1}\right)}^{2}+\text{s}.\text{e}.{\left(\text{ASIR}\right)}^{2}}}$$

We evaluated the ASIRs, standardized rate ratios (SRRs) based on patients’ histology information and various subsites within the bladder, including trigone (C67.0), dome (C67.1), lateral wall (C67.2), anterior wall (C67.3), posterior wall (C67.4), bladder neck (C67.5), ureteric orifice (C67.6), urachus (C67.7), overlapping lesions (C67.8), and unspecified parts (C67.9) of the bladder.

### 2.7 Ethics approval

The current study was approved by the Ethics Committee of Shahid Beheshti University of Medical Sciences (IR.SBMU.RETECH.REC.1400.554).

## 3 Results

### 3.1 Patient demographics and temporal trends in BCa (2006-2015)

A total of 51,379 BCa cases were identified during the study period (2006-2015), with a mean age of 65.10 ± 14.89 years, as presented in **Table 1**. Males comprised the majority of cases (81.97%). The overall trend revealed a gradual increase in BCa cases over the years, with the highest number (6,411) recorded in 2015.

**Table 1:** Patient demographics and trends over study period (2006-2015).

Variables	Frequency (%)/Mean ± SD
**No. of cases**	51379
**Age**	65.10 ± 14.89
Sex	
Male	42113 (81.97)
Female	9266 (18.03)
Year	
2006	4234 (8.24)
2007	4374 (8.51)
2008	4254 (8.28)
2009	3836 (7.47)
2010	5426 (10.56)
2011	5318 (10.35)
2012	6023 (11.72)
2013	5944 (11.57)
2014	5559 (10.82)
2015	6411 (12.48)

The patient characteristics were summarized for the years 2006 to 2015.

Regarding data quality, a robust 92.60% of cases (47,577) were substantiated by morphologically verified diagnosis (MV%), underscoring a high degree of diagnostic accuracy. Moreover, 5.21% of cases (2,494) relied on clinical diagnosis, while 7.40% (1,308) were classified as death certificate-only cases (DCO%), where the diagnosis was established posthumously.

### 3.2 Histological and topographical distribution of BCa

**Table 2** presents the histological and topographical distribution of bladder neoplasms. UCC was the predominant histological type, accounting for 85.52% of cases. Other notable histological types include AC at 1.64% and SCC at 0.83%. Regarding topographical distribution, “malignant neoplasm of the bladder, unspecified”, constitutes the majority (96.90%), followed by the “trigone of the bladder” (0.90%) and “lateral wall of the bladder” (0.70%).

**Table 2:** Histological and topographical distribution of bladder cancer.

Group	Levels	Frequency (Percentage)
Histology	AC	841 (1.64)
	Other	6046 (11.77)
	Sarcoma	28 (0.05)
	SCC	424 (0.83)
	Signet Ring Carcinoma	39 (0.08)
	Small Cell Carcinoma	27 (0.05)
	Spindle Cell Carcinoma	36 (0.07)
	UCC	43938 (85.52)
Topography	C67.0: Malignant neoplasm of trigone of bladder	461 (0.90)
	C67.1: Malignant neoplasm of dome of bladder	123 (0.20)
	C67.2: Malignant neoplasm of lateral wall of bladder	383 (0.70)
	C67.3: Malignant neoplasm of anterior wall of bladder	105 (0.20)
	C67.4: Malignant neoplasm of posterior wall of bladder	87 (0.20)
	C67.5: Malignant neoplasm of bladder neck	164 (0.30)
	C67.6: Malignant neoplasm of ureteric orifice	43 (0.10)
	C67.7: Malignant neoplasm of urachus	13 (0.00)
	C67.8: Overlapping lesion of bladder	210 (0.40)
	C67.9: Malignant neoplasm of bladder, unspecified	49790 (96.90)

UCC, urothelial cell carcinoma; SCC, squamous cell carcinoma; AC, adenocarcinoma.

### 3.3 Age-specific incidence rates (per 100,000 person-years) of BCa by sex

**Figure 1:**
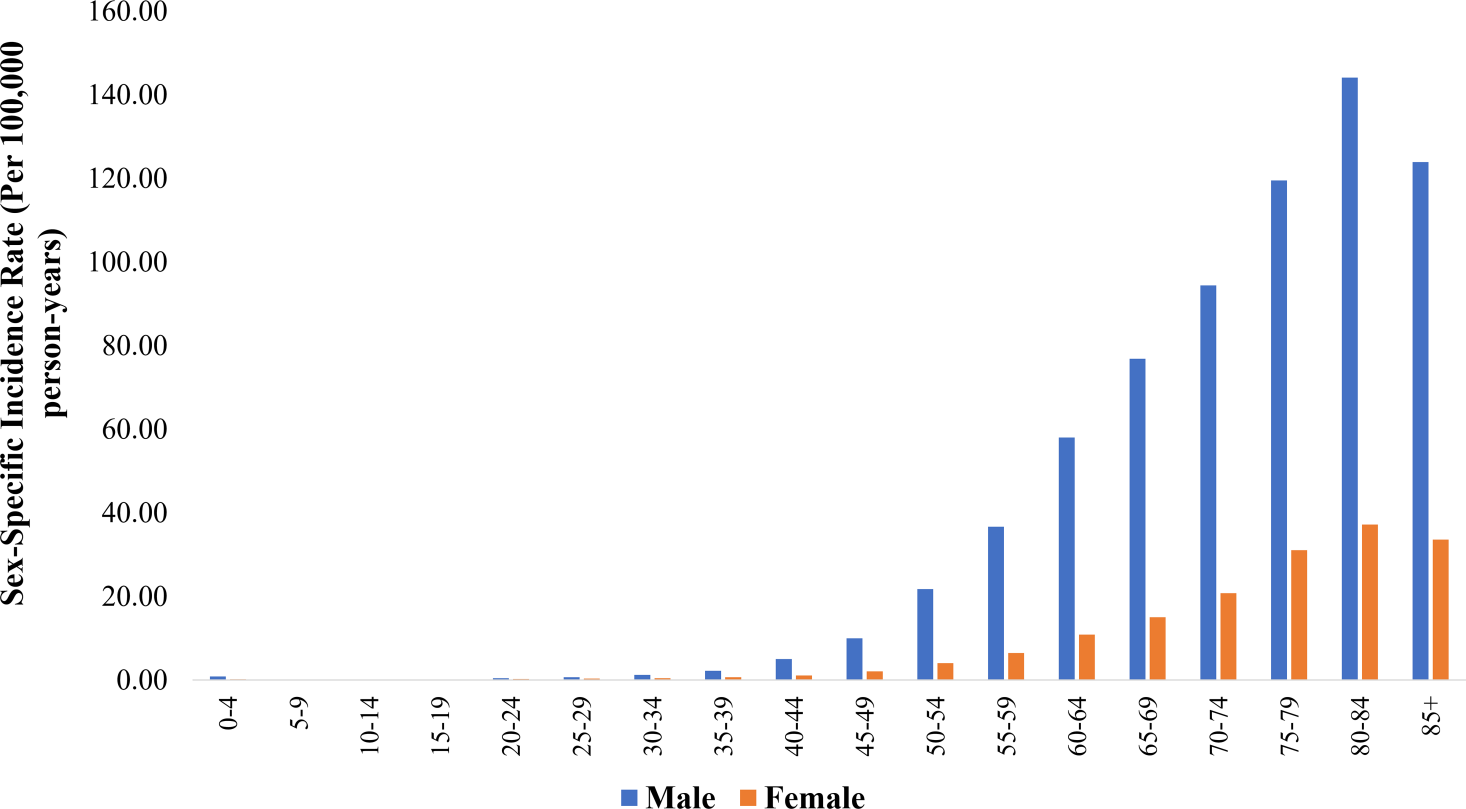
Sex-specific incidence rate (per 100,000 person-years) of bladder cancer across age groups.

**Figure 1** (and **Supplementary Table 1**) presents age-specific incidence rates for BCa, stratified by sex. Males showed persistently higher rates in all age groups. Particularly in the 50-54 and 55-59 age groups, males exhibited a significantly higher incidence rate than females (21.76 vs. 4.06 and 36.71 vs. 6.46, respectively). The sex disparity widened in older age groups, reaching a peak in the 80-84 category (males: 144.14 vs. females: 37.22). The increase in BCa incidence was particularly notable when comparing the age groups 40-44 to 80-84 years. Females experienced a nearly 33.5-fold rise in incidence, increasing from 1.11 to 37.22 per 100,000 person-years. In comparison, males exhibited a 28.6-fold increase, rising from 5.03 to 144.14 per 100,000 person-years.

### 3.4 ASIR of BCa (2006-2015): total and sex-specific analysis

**Table 3** (and **Supplementary Figure 1**) illustrates the temporal trends in ASIR (per 100,000 person-years) of BCa, categorized by histological types and sex (2006-2015). Across all years, the total ASIR remained relatively stable, ranging from 6.98 to 9.95. Notably, UCC consistently accounted for the highest incidence rates, varying from 6.34 to 8.23, with males constantly presenting higher rates than females. Additionally, specific histological types such as AC displayed varying patterns, and SCC showed a decreasing trend over time.

**Table 3:** Temporal trends in age-standardized incidence rates (per 100,000 person-years) of bladder cancer by histology types and sex (2006-2015).

		Total	AC	Other	Sarcoma	SCC	Signet Ring Carcinoma	Small Cell Carcinoma	Spindle Cell Carcinoma	UCC
2006	Total	8.77	0.13	0.33	0.00	0.11	0.00	0.00	0.00	8.19
	Male	14.13	0.19	0.54	0.01	0.16	0.01	0.00	0.00	13.21
	Female	3.17	0.07	0.11	0.00	0.05	0.00	0.00	0.00	2.94
2007	Total	8.68	0.12	0.34	0.00	0.11	0.00	0.00	0.00	8.11
	Male	14.00	0.17	0.52	0.01	0.13	0.01	0.00	0.00	13.17
	Female	3.20	0.07	0.15	0.00	0.08	0.00	0.00	0.00	2.89
2008	Total	8.09	0.12	0.46	0.01	0.09	0.01	0.00	0.00	7.40
	Male	13.18	0.17	0.66	0.00	0.15	0.01	0.01	0.00	12.18
	Female	2.92	0.07	0.26	0.01	0.02	0.00	0.00	0.00	2.56
2009	Total	6.98	0.09	0.46	0.00	0.07	0.01	0.01	0.00	6.34
	Male	11.50	0.13	0.71	0.00	0.10	0.01	0.01	0.01	10.52
	Female	2.48	0.04	0.22	0.00	0.03	0.01	0.00	0.00	2.17
2010	Total	9.48	0.32	1.07	0.00	0.09	0.01	0.00	0.01	8.23
	Male	15.49	0.51	1.57	0.00	0.15	0.02	0.00	0.02	13.61
	Female	3.57	0.14	0.58	0.01	0.04	0.00	0.00	0.00	2.92
2011	Total	9.01	0.16	1.31	0.00	0.07	0.01	0.00	0.01	7.46
	Male	14.88	0.27	2.11	0.00	0.11	0.01	0.01	0.01	12.35
	Female	3.25	0.06	0.52	0.00	0.02	0.00	0.00	0.00	2.65
2012	Total	9.95	0.12	1.54	0.00	0.07	0.01	0.01	0.01	8.18
	Male	16.67	0.22	2.50	0.01	0.12	0.01	0.01	0.01	13.80
	Female	3.31	0.03	0.60	0.00	0.02	0.00	0.00	0.01	2.64
2013	Total	9.53	0.14	1.54	0.01	0.05	0.01	0.00	0.01	7.77
	Male	15.63	0.22	2.34	0.01	0.07	0.01	0.00	0.02	12.94
	Female	3.50	0.06	0.73	0.00	0.03	0.00	0.00	0.00	2.67
2014	Total	8.62	0.11	1.42	0.00	0.05	0.00	0.01	0.00	7.02
	Male	14.19	0.15	2.13	0.00	0.06	0.00	0.02	0.00	11.81
	Female	3.31	0.03	0.60	0.00	0.02	0.00	0.00	0.01	2.64
2015	Total	9.64	0.11	1.51	0.01	0.06	0.01	0.01	0.01	7.93
	Male	15.95	0.16	2.31	0.01	0.08	0.01	0.02	0.01	13.34
	Female	3.43	0.06	0.70	0.00	0.04	0.00	0.00	0.01	2.61

UCC, urothelial cell carcinoma; SCC, squamous cell carcinoma; AC, adenocarcinoma.

As shown in **Table 4** (and **Supplementary Table 2**), the total ASIR for BCa was 8.92 (95% CI 8.84-9.00) per 100,000 person-years, with males demonstrating a substantially higher rate (14.64, 95% CI 14.50-14.79) compared to females (3.20, 95% CI 3.14-3.27). UCC dominated, with a total ASIR of 7.64 (95% CI 7.57-7.71) per 100,000 person-years, with higher rates in males (12.67, 95% CI 12.54-12.80) than females (2.61, 95% CI 2.55-2.67). Other histological types (AC, etc.) also showed varying ASIR patterns across sexes.

**Table 4:** Sex-based analysis of age-standardized incidence rates (per 100,000 person-years with 95% confidence interval) for bladder cancer histology types (2006-2015).

	2006-2015		
	Total	Male	Female
Total	8.92 (8.84-9.00)	14.64 (14.50-14.79)	3.20 (3.14-3.27)
AC	0.14 (0.13-0.15)	0.22 (0.20-0.24)	0.07 (0.06-0.07)
Other	1.04 (1.02-1.07)	1.61 (1.56-1.65)	0.48 (0.46-0.51)
Sarcoma	0.00 (0.00-0.01)	0.01 (0.00-0.01)	0.00 (0.00-0.00)
SCC	0.07 (0.07-0.08)	0.11 (0.10-0.12)	0.04 (0.03-0.04)
Signet Ring Carcinoma	0.01 (0.00-0.01)	0.01 (0.01-0.01)	0.00 (0.00-0.00)
Small Cell Carcinoma	0.00 (0.00-0.01)	0.01 (0.01-0.01)	0.00 (0.00-0.00)
Spindle Cell Carcinoma	0.01 (0.00-0.01)	0.01 (0.01-0.01)	0.00 (0.00-0.00)
UCC	7.64 (7.57-7.71)	12.67 (12.54-12.80)	2.61 (2.55-2.67)

ASIR, Age-standardized incidence rate; UCC, urothelial cell carcinoma; SCC, squamous cell carcinoma; AC, adenocarcinoma.

### 3.5 Sex disparities and temporal trends: SRR analysis for BCa by histological types (2006-2015)

**Table 5** (and **Supplementary Table 3**) present BCa SRRs (both male-to-female and 2011-2015 to 2006-2010), across various histological types. The overall male-to-female SRR was 4.57 (95% CI 4.48-4.67), indicating a significantly higher incidence in males compared to females. When examining specific histologies, the male-to-female SRR varied, with notable ratios for small cell carcinoma (27.86, 95% CI 7.15-108.48), UCC (4.85, 95% CI 4.74-4.96), and signet ring carcinoma (4.26, 95% CI 2.02-9.00), indicating substantial sex disparities. Considering the changes in incidence rates between two time periods (2006-2010 vs. 2011-2015), values ranged from 0.64 to 3.21, suggesting variations across histological types. Notably, small cell carcinoma (SRR 3.20, 95% CI 1.37-7.49) and spindle cell carcinoma (SRR 2.20, 95% CI 1.10-4.40) displayed increases in incidence rates during the latter period.

**Table 5:** Male to female and 2011-2015 to 2006-2010 standardized rate ratios for bladder cancer by histology.

Histology	Male to Female SRR	2011-2015 to 2006-2010 SRR
Total	4.57 (4.48-4.67)	1.11 (1.09-1.13)
AC	3.36 (2.89-3.92)	0.80 (0.70-0.92)
Other	3.34 (3.15-3.53)	2.70 (2.56-2.86)
Sarcoma	2.11 (0.96-4.64)	1.04 (0.49-2.22)
SCC	3.09 (2.50-3.82)	0.64 (0.53-0.78)
Signet Ring Carcinoma	4.26 (2.02-9.00)	0.78 (0.41-1.49)
Small Cell Carcinoma	27.86 (7.15-108.48)	3.21 (1.37-7.49)
Spindle Cell Carcinoma	4.02 (1.88-8.59)	2.20 (1.10-4.40)
UCC	4.85 (4.74-4.96)	1.01 (0.99-1.03)

UCC, urothelial cell carcinoma; SCC, squamous cell carcinoma; AC, adenocarcinoma; SRR, Standardized Rate Ratio.

## 4 Discussion

Our study investigated the incidence trends of BCa in Iran from 2006 to 2015, stratified by sex, age, histology subtypes, and topography. We observed a modest increase in the ASIR of BCa overall and among males. Notably, males consistently exhibited a significantly higher ASIR compared to females, with a ratio of approximately 4.5:1. BCa incidence displayed a progressive rise across older age groups, particularly those aged 40-44 to 80-84 years, with the increase being more pronounced in females (approximately 34-fold) compared to males (approximately 29-fold). Men were disproportionately affected by both UCC and AC, with rates approximately 5 and 3 times higher than females, respectively. Papillary TCC and TCC not otherwise specified (NOS) were the predominant histological subtypes, and “malignant neoplasm of the bladder, unspecified” constituted over 95% of BCa topography classifications.

This retrospective cohort study using data from the INCR has inherent limitations. Selection bias, information bias, and confounding are potential concerns in studies using previously registered data (20). We included all eligible individuals consecutively throughout the defined period to minimize selection bias and maximize coverage of BCa cases in Iran. Data quality checks and corrections were implemented to address information bias. However, the retrospective design limits our ability to fully control for confounding variables and potential changes in diagnostic practices over time. Age and sex-specific analyses were performed, but residual confounding remains a possibility. Additionally, the study’s reliance on historical data up to March 20, 2015, introduces a temporal limitation, as it may not capture recent developments in diagnostic techniques or changes in cancer reporting practices. These limitations necessitate a cautious interpretation of the findings. Additionally, geographic information regarding the province of residence for patients was not consistently recorded in the dataset. This limitation prevented us from calculating and presenting ASIRs across provinces, an important area for future studies that should aim to capture regional variations in bladder cancer incidence with more precise data collection. Despite these limitations, our study offers a unique contribution by extending beyond the simple analysis of BCa trends. We conducted a comprehensive analysis of BCa pathology by concurrently examining both histological subtypes and topographical distribution within the same time period. To our knowledge, this is the first study in Iran to concurrently explore BCa trends, histology, and topography over a decade. This unique approach gives us a more complete picture of how BCa characteristics and distribution are changing within the Iranian population. Future studies employing prospective designs with more comprehensive data collection methods are warranted to provide a more robust understanding of BCa epidemiology and pathology in Iran.

The overall ASIR found in this study was 8.92 per 100,000, which was exceeding the global ASIR (6.69 – 7.11) (21), but lower than rates in the UK (16.9) and Australia (approximately 12) (22, 23). The observed differences might be both due to the differences in geographical distribution of the disease or the presence of dedicated urological anatomical pathologists in different centers, which can considerably increase the detection rate of BCa, especially the rare variants (24–26).

The 2015 ASIR for BCa reported in this study (around 10 per 100,000) aligns with findings from recent studies by Nowroozi et al. (2024) and Mousavian et al. (2023) (2, 27). However, a higher value was observed in this study compared to the ASIR reported by the GBD study for Iran, which reported a value of 6.02 per 100,000 persons (3). An analysis of the ASIR trend for BCa revealed a fluctuating and slightly increasing pattern in this study, similar to the trend observed in the GBD analysis (3). In contrast, a steeper increasing trend was reported by Nowroozi et al. (2024) and Mousavian et al. (2023) (2, 27). This discrepancy suggests potential differences in data quality of INCR, particularly in the earlier years of the study period, and may indicate the underrepresentation of new BCa cases in the aforementioned recent studies, likely due to data incompleteness or other factors impacting data collection efficiency. Notably, changes in the registration practices of the INCR between the two time periods (2006–2010 and 2011–2015) may also have contributed to the observed differences in BCa incidence, highlighting the potential impact of data collection improvements rather than a true increase in BCa cases. Our study benefited from utilizing a more comprehensive and complete version of INCR data over the entire study duration, providing a robust foundation for our findings.

SEER and Cancer Research UK are renowned for their high-quality cancer registries. SEER reported the ASIR of BCa from 2005 to 2015, ranging from 20.87 to 19.71 per 100,000 persons (28), while Cancer Research UK documented approximately similar rates of 20 to 17 per 100,000 persons during the same timeframe (29), both indicating a slight downward trend in BCa incidence. However, the rates reported by these organizations are higher than those obtained in our study, which ranged from 14.13 to 15.95 per 100,000 persons with a slight increasing trend observed. Furthermore, our study revealed higher rates compared to the Eastern Mediterranean region (defined by WHO) and Lower-Middle Income Countries (defined by World Bank) where Iran is situated- with values ranging from 7.77 to 8.71 and 2.92 to 3.06 per 100,000 persons, respectively (3). The difference in ASIR between Iran and other regions could be due to several factors, including variations in lifestyle habits such as smoking prevalence, dietary patterns, environmental exposures, access to healthcare and diagnostic services, as well as differences in data collection and reporting methodologies across regions.

Our study revealed a significantly higher ASIR of BCa among males compared to females, with a ratio of approximately 4.5:1, consistent with findings from recent studies (1, 2, 4, 30, 31). Several studies shed light on potential explanations for this disparity. Alcala et al. (2023) reported a higher prevalence of opium use and smoking among males contributing to BCa risk, with 8% of male cases attributed to opium use compared to 2% in females. Similarly, Cigarette smoking was directly associated with 12% of BCa cases in smokers compared to 2% in non-smokers, indicating a significant role of smoking in causing this disease (5). Rasti et al. (2023) investigated sex-specific factors influencing BCa by identifying six key genes (IGF2, CCL5, ASPM, CDC20, BUB1B, CCNB1) linked to these differences. Their findings suggest that certain genes exhibit variations in activity based on sex, potentially affecting BCa risk and prognosis (32). Doshi et al. (2023) emphasized the role of biological factors in the observed sex gap. They highlighted the influence of circulating hormone levels, hormone receptor expression, and genetic/epigenetic alterations on BCa development and outcomes in males and females (33). Additionally, Lutz et al. (2021) proposed several potential factors contributing to the sex disparity in BCa incidence, including androgen-induced immunosuppression, androgen-induced urothelial and neoplastic proliferation, increased male exposure to carcinogens, and the protective effects of X chromosome genes in females (34). These studies collectively highlight the multifaceted nature of sex disparities in BCa, emphasizing the importance of considering biological, genetic, and environmental factors for a comprehensive understanding of BCa epidemiology.

Our findings revealed a significant increase in BCa incidence beyond the age of 40-44 years, with the peak incidence observed among individuals aged 80-84 years. In this older age group, females exhibited a 30-fold increase, and males a 25-fold increase, in BCa incidence compared to the middle-aged group (40-44 years). This rising trend aligns with studies by Shokri Varniab et al. (2023) and Hadavandsiri et al. (2023), reporting incidence rates exceeding 47 and 100 per 100,000 persons for patients aged 70 years and above in Iran, respectively (35, 36). Specifically, Hadavandsiri et al. (2023) study indicated an increase in BCa incidence beginning after the age of 40-44 years (36). Consistent trends in BCa incidence rates with advancing age beyond 40-44 years were also observed in the SEER study and Cancer Research UK data. The higher likelihood of cancer development in older individuals may be attributed to prolonged exposure to carcinogenic agents, DNA damage accumulation, tumor suppressor gene mutations, oncogenic activation, and diminished immune response (37). However, advancements in preventive and control measures are expected to delay cancer onset, resulting in a higher proportion of cancer incidence among the older adult compared to other age groups (38).

BCa exhibits a spectrum of histological subtypes, categorized as urothelial or non-urothelial. Urothelial carcinoma itself encompasses diverse variants, each with unique characteristics like metastatic potential, immunotherapy target expression, and response to treatment. This heterogeneity poses diagnostic and therapeutic challenges due to the rarity of some variants, potentially leading to missed diagnoses and suboptimal management. While the prognosis for each variant requires further investigation, certain subtypes like micropapillary, sarcomatoid, and small cell carcinomas are known to have a poorer outcome compared to those with squamous or glandular differentiation. Treatment recommendations, including neo-adjuvant chemotherapy and radiation therapy, depend heavily on the specific histological variant (11). Non-urothelial histologies, whether pure or mixed with UCC, generally have a worse prognosis. However, limited information on these subtypes often leads to a broad classification of “non-urothelial,” overlooking crucial clinical and biological differences (12). Our study, the most recent of its kind in Iran, investigated the distribution of these histological groups. UCC and AC emerged as the most prevalent BCa histological types, consistently observed throughout the study period. Papillary TCC and NOS TCC were the predominant histological subtypes within UCC. Our findings regarding the dominance of UCC are consistent with previous studies by Rafiemanesh et al. (2015) on Iranian patients and Patel et al. (2018) using SEER data. However, unlike these studies where SCC and carcinoma NOS ranked second and third, respectively, AC emerged as the second most prevalent subtype in our study (12, 13). This discrepancy suggests potential geographic variations in BCa subtype distribution, possibly influenced by environmental exposures or diagnostic practices. For instance, chronic schistosomiasis infection, a risk factor for SCC, might be less prevalent in Iran (39). Additionally, advancements in diagnostic tools could improve the identification of AC histology type.

## 5 Conclusion

This study investigated BCa incidence trends in Iran from 2006 to 2015, revealing a modest increase both overall and among males. Notably, males consistently exhibited a significantly higher ASIR compared to females. BCa incidence displayed a progressive rise with age, particularly in older individuals. UCC and AC were the most prevalent histological subtypes, with papillary TCC and TCC NOS being predominant. While limitations inherent to the retrospective design necessitate cautious interpretation, our findings provide valuable insights into BCa epidemiology in Iran. The observed sex disparity aligns with previous studies, potentially explained by factors like smoking and opium use prevalence, genetic predisposition, and hormonal influences. The age-related trends suggest a higher incidence among the older adult, likely due to prolonged exposure to carcinogens and age-related declines in cellular repair mechanisms. Our study identified UCC and AC as the most frequent BCa histological types, with a discrepancy in AC prevalence compared to some prior studies, possibly reflecting geographic variations in risk factors or diagnostic practices. Future prospective studies with comprehensive data collection are warranted to elucidate the underlying mechanisms of BCa development and inform preventive strategies in Iran. Additionally, further research is needed to explore the specific contributions of lifestyle habits, environmental exposures, and genetic susceptibility in the context of BCa risk and prognosis in the Iranian population.

## Data Availability

The data analyzed in this study is subject to the following licenses/restrictions: The datasets generated and analyzed during the current study are not publicly available due to them containing information that could compromise participant privacy. However, anonymized data may be made available by request to INCR, the nationwide registry maintained by the Ministry of Health and Medical Education. Requests to access these datasets should be directed to profmeakbari@gmail.com. Requests to access these datasets should be directed to Mohammad Esmaeil Akbari, profmeakbari@gmail.com.
