# Supplementary material for: Incidence trends, histological subtypes, and topographical distribution of bladder cancer in Iran: a study based on the Iranian National Cancer Registry during 2006-2015
Source: Front Oncol. 2024 Oct 8;14:1423968. doi: 10.3389/fonc.2024.1423968 (PMC11493537; doi:10.3389/fonc.2024.1423968)
Supplement: Supplementary file 1 [file DataSheet1.docx]

**Supplementary File**

Supplementary Table 1. Sex-Specific Incidence (Per 100,000 person-years) of Bladder Cancer Across Age Groups

| **Age Group** | **Male** | **Female** | **Total** |
| --- | --- | --- | --- |
| 0-4 | 0.86 | 0.20 | 0.54 |
| 5-9 | 0.09 | 0.05 | 0.07 |
| 10-14 | 0.06 | 0.03 | 0.04 |
| 15-19 | 0.14 | 0.05 | 0.10 |
| 20-24 | 0.43 | 0.25 | 0.34 |
| 25-29 | 0.68 | 0.33 | 0.51 |
| 30-34 | 1.23 | 0.45 | 0.85 |
| 35-39 | 2.22 | 0.68 | 1.46 |
| 40-44 | 5.03 | 1.11 | 3.10 |
| 45-49 | 9.97 | 2.10 | 6.08 |
| 50-54 | 21.76 | 4.06 | 12.94 |
| 55-59 | 36.71 | 6.46 | 21.44 |
| 60-64 | 58.00 | 10.89 | 33.67 |
| 65-69 | 76.87 | 15.04 | 44.95 |
| 70-74 | 94.43 | 20.77 | 57.78 |
| 75-79 | 119.53 | 31.06 | 77.15 |
| 80-84 | 144.14 | 37.22 | 92.10 |
| 85+ | 123.92 | 33.59 | 80.12 |

Supplementary Table 2. Age-Standardized Incidence Rates (Per 100,000 Person-Years With 95% Confidence Interval) for Bladder Cancer Histology Types by Sex and Year (2006-2015)

|  | **2006-2010** | | | **2011-2015** | | | **2006-2015** | | |
| --- | --- | --- | --- | --- | --- | --- | --- | --- | --- |
|  | **Total** | **Male** | **Female** | **Total** | **Male** | **Female** | **Total** | **Male** | **Female** |
| Total | 8.40 (8.29-8.51) | 13.66 (13.46-13.86) | 3.07 (2.97-3.17) | 9.35 (9.24-9.46) | 15.47 (15.27-15.67) | 3.32 (3.23-3.41) | 8.92 (8.84-9.00) | 14.64 (14.50-14.79) | 3.20 (3.14-3.27) |
| AC | 0.16 (0.14-0.17) | 0.24 (0.21-0.27) | 0.08 (0.06-0.09) | 0.13 (0.12-0.14) | 0.20 (0.18-0.22) | 0.05 (0.04-0.07) | 0.14 (0.13-0.15) | 0.22 (0.20-0.24) | 0.07 (0.06-0.07) |
| Other | 0.54 (0.51-0.57) | 0.81 (0.76-0.86) | 0.27 (0.24-0.30) | 1.46 (1.42-1.51) | 2.28 (2.20-2.35) | 0.66 (0.62-0.70) | 1.04 (1.02-1.07) | 1.61 (1.56-1.65) | 0.48 (0.46-0.51) |
| Sarcoma | 0.00 (0.00-0.01) | 0.01 (0.00-0.01) | 0.00 (0.00-0.01) | 0.00 (0.00-0.01) | 0.01 (0.00-0.01) | 0.00 (0.00-0.00) | 0.00 (0.00-0.01) | 0.01 (0.00-0.01) | 0.00 (0.00-0.00) |
| SCC | 0.09 (0.08-0.10) | 0.14 (0.12-0.16) | 0.04 (0.03-0.06) | 0.06 (0.05-0.07) | 0.09 (0.07-0.10) | 0.03 (0.02-0.04) | 0.07 (0.07-0.08) | 0.11 (0.10-0.12) | 0.04 (0.03-0.04) |
| Signet Ring Carcinoma | 0.01 (0.00-0.01) | 0.01 (0.01-0.02) | 0.00 (0.00-0.01) | 0.01 (0.00-0.01) | 0.01 (0.00-0.01) | 0.00 (0.00-0.00) | 0.01 (0.00-0.01) | 0.01 (0.01-0.01) | 0.00 (0.00-0.00) |
| Small Cell Carcinoma | 0.00 (0.00-0.00) | 0.00 (0.00-0.01) | 0.00 (0.00-0.00) | 0.01 (0.00-0.01) | 0.01 (0.01-0.02) | 0.00 (0.00-0.00) | 0.00 (0.00-0.01) | 0.01 (0.01-0.01) | 0.00 (0.00-0.00) |
| Spindle Cell Carcinoma | 0.00 (0.00-0.01) | 0.01 (0.00-0.01) | 0.00 (0.00-0.00) | 0.01 (0.01-0.01) | 0.01 (0.01-0.02) | 0.00 (0.00-0.01) | 0.01 (0.00-0.01) | 0.01 (0.01-0.01) | 0.00 (0.00-0.00) |
| UCC | **7.59 (7.48-7.69)** | **12.44 (12.25-12.64)** | **2.67 (2.58-2.76)** | **7.68 (7.58-7.77)** | **12.86 (12.68-13.04)** | **2.57 (2.49-2.65)** | **7.64 (7.57-7.71)** | **12.67 (12.54-12.80)** | **2.61 (2.55-2.67)** |

Abbreviation: urothelial cell carcinoma (UCC), squamous cell carcinoma (SCC), adenocarcinoma (AC)

Supplementary Table 3. Male to Female and 2011-2015 to 2006-2010 Standardized Rate Ratios for Bladder Cancer by Histology

| Histology | **Male to Female SRR** | | | **2011-2015 to 2006-2010 SRR** |
| --- | --- | --- | --- | --- |
|  | **2006-2010** | **2011-2015** | **2006-2015** |  |
| Total | 4.4 (4.3-4.6) | 4.7 (4.5-4.8) | 4.57 (4.48-4.67) | 1.11 (1.09-1.13) |
| AC | 3.0 (2.4-3.7) | 3.8 (3.0-4.7) | 3.36 (2.89-3.92) | 0.80 (0.70-0.92) |
| Other | 3.0 (2.7-3.4) | 3.5 (3.2-3.7) | 3.34 (3.15-3.53) | 2.70 (2.56-2.86) |
| Sarcoma | 1.5 (0.5-4.7) | 2.9 (1.0-8.5) | 2.11 (0.96-4.64) | 1.04 (0.49-2.22) |
| SCC | 3.1 (2.3-4.1) | 3.1 (2.2-4.3) | 3.09 (2.50-3.82) | 0.64 (0.53-0.78) |
| Signet Ring Carcinoma | 4.1 (1.5-11.2) | 4.8 (1.5-14.7) | 4.26 (2.02-9.00) | 0.78 (0.41-1.49) |
| Small Cell Carcinoma | #DIV/0! | 22.9 (5.3-99.8) | 27.86 (7.15-108.48) | 3.21 (1.37-7.49) |
| Spindle Cell Carcinoma | 8.8 (1.6-48.7) | 3.3 (1.4-7.7) | 4.02 (1.88-8.59) | 2.20 (1.10-4.40) |
| UCC | 4.7 (4.5-4.8) | 5.0 (4.9-5.2) | 4.85 (4.74-4.96) | 1.01 (0.99-1.03) |

Abbreviation: urothelial cell carcinoma (UCC), squamous cell carcinoma (SCC), adenocarcinoma (AC), Standardized Rate Ratios (SRR)

Supplementary Table 4. Distribution of Histology Subtypes in Bladder Cancer

| **Histology subtypes** | **Frequency (Percentage)** |
| --- | --- |
| Adenocarcinoma, NOS | 553 (1.08) |
| Alveolar rhabdomyosarcoma | 2 (0.00) |
| Carcinoma, NOS | 555 (1.08) |
| Carcinoma, undiff., NOS | 151 (0.29) |
| Carcinosarcoma, NOS | 5 (0.01) |
| Embryonal rhabdomyosarcoma | 19 (0.04) |
| Fibromatous neoplasms | 2 (0.00) |
| Folic. & marginal lymph, NOS | 3 (0.01) |
| Giant & spindle cell carcinoma | 51 (0.10) |
| Granular cell carcinoma | 29 (0.06) |
| Hodgkin lymphoma | 1 (0.00) |
| Hodgkin lymphoma, nod. scler. | 3 (0.01) |
| Malignant lymphoma, NOS | 17 (0.03) |
| Medullary carcinoma, NOS | 1 (0.00) |
| Ml, large b-cell, diffuse | 10 (0.02) |
| Ml, small b-cell lymphocytic | 2 (0.00) |
| Mucinous adenocarcinoma | 61 (0.12) |
| Myomatous neoplasms | 12 (0.02) |
| Neoplasm | 3623 (7.05) |
| Papillary adenocarcinoma, NOS | 201 (0.39) |
| Papillary carcinoma, NOS | 1405 (2.73) |
| Papillary transitional cell carcinoma | 25754 (50.13) |
| Rhabdomyosarcoma, NOS | 12 (0.02) |
| Sarcoma, NOS | 45 (0.09) |
| Signet ring cell carcinoma | 39 (0.08) |
| Small cell carcinoma, NOS | 27 (0.05) |
| Solid carcinoma, NOS | 3 (0.01) |
| Squamous cell carcinoma, NOS | 449 (0.87) |
| T-cell lymphomas | 1 (0.00) |
| Transitional cell carcinoma, NOS | 18244 (35.51) |
| Unknown | 99 (0.19) |

Supplementary Figure 1. Temporal Trends in Age-Standardized Incidence Rates of Bladder Cancer from 2005 to 2016
